# Peptidome analysis of cerebrospinal fluid in neonates with hypoxic-ischemic brain damage

**DOI:** 10.1186/s13041-020-00671-9

**Published:** 2020-10-02

**Authors:** Xuewen Hou, Zijun Yuan, Xuan Wang, Rui Cheng, Xiaoguang Zhou, Jie Qiu

**Affiliations:** Department of Newborn Infants, Children’s Hospital of Nanjing Medical University, Nanjing, 210008 Jiangsu China

**Keywords:** Peptidomics, Cerebrospinal fluid, Neonates, Hypoxic-ischemic brain damage, Pyroptosis

## Abstract

Hypoxic-ischemic brain injury (HIBD) causes neonatal death and serious neurological disability; however, there are currently no promising therapies for it excepting cooling. Therefore, in this study, we used peptidome analysis to identify differentially expressed peptides in cerebrospinal fluid (CSF) of neonates with HIBD or controls, which may give a foundation for finding new promising drugs of neonatal HIBD. CSF samples were collected from neonates with HIBD (n = 4) or controls (n = 4). ITRAQ LC–MS/MS was used to identify differentially expressed peptides between two groups. A total of 35 differentially expressed peptides from 25 precursor proteins were identified. The 2671.5 Da peptide (HSQFIGYPITLFVEKER), one of the down-regulated peptides in neonatal HIBD, is a fragment of heat shock protein 90-alpha (HSP90α/HSP90AA1). Results of bioinformatics analysis showed that HSP90α/HSP90AA1 was located in the protein–protein interaction (PPI) network hub and was involved in the NOD-LIKE receptor (NLR) signaling pathway. This peptide, we named it Hypoxic-Ischemic Brain Damage Associated Peptide (HIBDAP), is a hydrophilic peptide with high stability and has a long half-life of 3.5 h in mammalian reticulocytes. It was demonstrated that TAT-HIBDAP could successfully enter PC12 cells and further into the nucleus. After HIBDAP pretreatment and 6 h of OGD treatment, low concentrations of HIBDAP increased the survival rate of cells, except 40 μM had a toxic effect. Safe concentrations of HIBDAP reduced pyroptosis of PC12 cells under OGD, except 20 μM had no effect, by suppressing expressions of NLRP3, ASC and Caspase-1 except NLRP1. The results of our study identified the characterization and expression profiles of peptides in CSF of neonatal HIBD. Several meaningful peptides such as HIBDAP may play significant roles in neonatal HIBD and provide new therapeutic targets for neonatal HIBD.

## Introduction

Hypoxic ischemic brain injury (HIBD) which could cause neuronal and white matter injury [1] is one of the most common causes of neonatal death. HIBD accounts for 23% of global infant mortality [2] and up to 25% survivors have permanent neuropsychological deficits, including cerebral palsy, learning and behavioral disabilities, epilepsy, and so on [3–5]. However, until now, there are few effective treatments for HIBD except the hypothermia therapy [6–8]. Therefore, looking for new HIBD therapeutic methods is urgently needed.

Peptides, low molecular weight fractions of proteins, play significant roles in physiological processes [9], such as gene expression, substance metabolism and other processes [10]. In recent years, peptides as drugs have attracted much attention because they are simpler and cheaper than protein-based drugs. Meanwhile, compared to small-molecule drugs, they are safer and more effective because they are highly selective and effective signal molecules. Until now, over 60 peptide drugs have reached the market for the benefit of patients, approximately 140 peptide drugs were in clinical trials and more than 500 therapeutic peptides were in preclinical studies [11]. However, researches of peptide drugs are mainly in the fields of metabolic diseases, tumors and cardiovascular diseases; there were few reports in neonatal HIBD.

It is notable that some peptides have been found to play important roles in ischemic brain injury. TFP5, a modified 24-aa peptide (lys254-ala277) derived from p35, could significantly reduce the cerebral infarction area after ischemic stroking [12]. Another peptide named DEETGE-CAL-Tat peptide also plays a neuroprotective role and maintains cognitive function in ischemic brain injury [13]. Particularly, several recent studies have demonstrated that cationic arginine-rich peptides (CARPs), which include many cell-penetrating peptides [e.g., transactivator of transcription (TAT) and poly-arginine-9 (R9; 9-mer of arginine)], possess intrinsic neuroprotective properties in perinatal hypoxic-ischemic [14]. Therefore, it is interesting to find useful peptides in neonatal HIBD and may provide new treatments for it in the clinic.

Peptidome, an emerging proteome technique, is a qualitative and quantitative analysis of peptides in cells, tissues or body fluids. With the development of instrument performance, liquid chromatography–tandem mass spectrometry (LC–MS/MS) with high detection sensitivity and high throughput can detect low abundance proteins and any types of proteins, such as membrane proteins, nuclear proteins, extracellular proteins and so on. So, in this study, we used LC–MS/MS to analyze differentially expressed peptides between neonatal HIBD and normal control.

For diseases of the central nervous system (CNS), CSF is a valuable source of diagnosis biomarkers, disease mechanisms and novel therapy targets [15]. Large amounts of peptides have been found in CSF [15, 16] and some of them are related to neurodegenerative diseases such as Alzheimer disease [17]. Therefore, peptidomic analysis of CSF from neonatal HIBD may give new clues to find new treatments of neonatal HIBD and has not been reported until now.

In brief, in this study, we aim to identify differentially expressed peptides in CSF of neonatal HIBD using LC–MS/MS. Through studying the function of one of these peptides, we hope to find new therapeutic peptide for neonatal HIBD.

## Methods

### Sample collection

Four HIBD infants from neonatal intensive care unit (NICU) of Children’s Hospital of Nanjing Medical University were selected. Inclusion criteria included (1) term infants with acute fetal distress (prolonged resuscitation need, and/or cord pH < 7.0, and/or Apgar score at 5 min < 5); (2) appearing neurological complication; (3) clear brain injury which diagnosed by magnetic resonance imaging (MRI) or computed tomography (CT). Exclusion criteria included serious brain injuries caused by infection, intracranial hemorrhage, genetic metabolic diseases or others. Control CSF samples were obtained from four matched infants without known neurological disease who required diagnostic lumbar punctures for routine sepsis evaluation. Table 1 shows the demographic characteristics of the neonates. All CSF samples were harvested within 24 h after birth and centrifuged at 3000 rpm for 10 min to acquire supernatants which were then stored in liquid nitrogen with protease inhibitor cocktail (Complete mini EDTA-free, Med Chem Express, USA). This study was approved by the ethics committee of Children’s Hospital of Nanjing Medical University and achieved agreements from infants’ parents.

**Table 1.** Clinical and demographic characteristics of neonates

	Control group (n = 4)	HIBD group (n = 4)
Sex (male/female)	2/2	1/3
Multiple births (%)	0 (0)	0 (0)
Gestational age (weeks)	38.9 ± 1.0	39.1 ± 1.2
Birth weight (g)	3400 ± 451	3540 ± 326
Cesarean section (%)	1 (25)	0 (0)
1-min APGAR	10 ± 0	3.5 ± 2.3
5-min APGAR	10 ± 0	5.1 ± 2.1

Data are presented as mean ± SD

APGAR indicates appearance, pulse, grimace, activity, respiration

### Sample treatment

All CSF samples were grinded under liquid nitrogen conditions, mixed with protein lysate (7 M urea, 2 M thiourea, 4% SDS, 40 mM Tris-HCl, pH8.5), 1 mM phenyl methyl sulfonyl fluoride (PMSF), 2 mM ethylene diamine tetraacetic acid (EDTA) and 10 mM dithiothreitol (DTT). The mixtures were ultra-sounded on ice for 10 min and then centrifuged at 12,000*g*, 4 °C for 30 min to collect liquid supernatant. Equal amounts of proteins were treated by reductive alkylation. Then, equal amounts of proteins were ultrafiltrated using molecular weight cut-off (MWCO) filters (Millipore, USA) of 10 kDa according to the manufacturer's recommendation. The flow-through from the filters containing peptide fractions was recycled, desalted, concentrated using C18 solid phase extraction (SPE) (Strata™-X, 33 μm, 2 g/20 mL, Phenomenex), and finally lyophilized.

### Labelling

Peptide samples were dissolved in 0.5 M Triethylammonium bicarbonate (TEAB) for peptide labeling after desalting. All peptides from samples were labeled with iTRAQ-8 standard kit (AB SCIEX Inc., Framingham, MA, USA). Then, the labeled samples were fractionated using a high-performance liquid chromatography (HPLC) system (Thermo DINOEX Ultimate 3000, USA) with a Durashell C18 (5 μm, 100A, 4.6 × 250 mm), and 12 fractions were collected.

### SDS-PAGE

The protein samples and protein marker (Fermentas, St. Leon-Rot, Germany) were subjected to 12% sodium dodecyl sulfate-polyacrylamide gel (SDS-PAGE), and protein bands were visualized by Coomassie brilliant blue staining.

### Liquid chromatography/mass spectrometry (LC–MS/MS)

Reverse-phase HPLC–ESI–MS/MS was used to analyze the fractionated samples. The data were collected and identified using the TripleTOF 5600 plus mass spectrometer with the Eksigent Ultra Plus nano-LC 2D HPLC system (AB SCIEX, USA). The freeze-dried peptide samples dissolving in 2% acetonitrile (ACN)/0.1% formic acid (FA) were loaded to a C18 trap column (5 µm, 100 μm × 20 mm, LC packings) and separated using the C18 analytical column (3 µm, 75 µm × 150 mm, LC packings) over a 90 min gradient at 300 nL/min. The two mobile phases of contained A, 2% ACN/98% of 0.1% FA (v/v) in water and B, 98% ACN/2% of 0.1% FA (v/v) in water.

For information-dependent acquisition (IDA), MS1 were scanned in 250 ms, and MS/MS of 30 precursor ions were scanned in 50 ms. MS1 spectra were collected in the range of 350–1500 m/z, and MS/MS spectra were collected in the range of 100–1500 m/z. Precursor ions were excluded from reselection for 15 s. For MS analysis, Proteinpilot™ database search engine (V4.5, AB SCIEX, USA) was used to protein identification based on the MS/MS spectra data which provides an automatic mass recalibration of the data and obtains more data.

### Bioinformatics analysis

The LC–MS/MS data were searched using the Mascot search engine (Matrix science) (https://www.matrixscience.com) against the SwissProt sequence database with the *Homo sapiens* subset including the following variable modifications: phosphorylation, amidation, deamidation, pyroglutamic acid, oxidation, acetylation, sulfation, oxidized and reduced cysteines. Gene ontology (GO) and kyoto encyclopedia of genes and genomes (KEGG) analysis were used to study potential functions of differentially expressed peptides and their precursor proteins. The protein–protein interaction networks were mapped using STRING database (https://string-db.org/) and UniProt database (https://www.uniprot.org/). The properties of peptides were checked through the website https://www.expasy.org and https://smart.embl-heidelberg.de/.

### Cell culture and treatment

PC12 rat pheochromocytoma cells were obtained from American Type Culture Collection (Rockville, MD, USA) and cultured in RPMI 1640 culture medium supplemented with 10% v/v horse serum (HS), 5% v/v fetal bovine serum (FBS) and appropriate antibiotics in a humidified chamber (5% CO_2_ and 37 °C), all of which were purchased from Invitrogen Life Technologies (Carlsbad, CA, USA).

For the induction of oxygen and glucose deprivation (OGD), cells were switched to RPMI 1640 without glucose after washing twice with glucose-free RPMI 1640. TAT-HIBDAP (YGRKKRRQRRR-HSQFIGYPITLFVEKER) and the FITC tagged TAT-HIBDAP (Shanghai Science Peptide Biological Technology Co., Ltd., China) were dissolved in sterile water and added to the glucose-free culture medium. After 1 h, cells were placed into an atmosphere of 2% O_2_, 5% CO_2_ and 93% N_2_ at 37 °C for 6 h. Control cells were maintained in glucose-containing RPMI 1640 and incubated in a normoxic incubator for the same time.

### Cell viability assay

Cell viability was measured using cell counting kit-8 (CCK-8) assay (Dojindo Molecular Technologies, Tokyo, Japan) according to the manufacturer’s protocol. Cells were inoculated into a 96-well plate (1 × 10^4^ cells/hole). Cells in 90 μL glucose-free culture medium of each well were added with 10 μL CCK-8 solution and incubated for another 1 h in hypoxic environment. The absorbance was measured at 450 nm using a Microplate Reader (Themo scientific, Vantaa, Finland). All experiments were independently repeated three times.

### Annexin V-fluorescein isothiocyanate (FITC) Assay

Cells were labeled by FITC-coupled Annexin V (Annexin V-FITC) (BD Biosciences, NJ, USA) for detection of phosphatidylserine exposure and by propidium iodide (PI) for observation of the loss of membrane integrity. Cells were harvested by trypsinization and washed twice with ice-cold phosphate buffer saline (PBS). Then, cells were resuspended in 1× binding buffer at a concentration of 1 × 10^6^ cells/mL. Transferred 100 μL of the solution (1 × 10^5^ cells) to a 5 mL culture tube and stained with 5 μL Annexin V-FITC and 5 μL propidium iodide (PI). The suspension was incubated at room temperature in the dark for 15 min. Added 400 μL of 1× binding buffer to each tube. FACS Calibur Flow Cytometer (BD Biosciences, NJ, USA) was used to distinguish cells and the results were analyzed by FlowJo software (Tree Star Corp, Ashland, OR). All experiments were independently repeated three times.

### Transmission electron microscopic examination

PC12 cells were fixed in 2.5% glutaraldehyde and rinsed with 0.1 mol/L phosphate-buffered saline (PBS). Samples were placed in 1% osmium acid at 4 °C for 4 h. After that, they were dehydrated in ethanol, embedded in the embedding agent, and stained with uranyl acetate and lead citrate. Finally, samples were examined under a transmission electron microscope (JEM-1400, JEOL, Japan).

### Quantitative real-time polymerase chain reaction (qRT-PCR)

Total RNA of cells was extracted using Trizol reagent (Invitrogen, Carlsbad, CA) according to the manufacturer’s instruction. HiScript^®^ II Q RT SuperMix for qPCR (Vazyme Biotech, Nanjing, China) was used for reverse transcription of mRNA following the manufacturer’s instruction. The RT thermal cycle program was as follows: 50 °C for 15 min and 85 °C for 5 s. The qPCR step was performed using a 7900HT Fast Real-Time PCR system with a TaqMan^®^ MicroRNA Assay kit (Applied Biosystems, CA, USA) as the following conditions: 95 °C for 5 min, followed by 40 cycles (95 °C for 10 s and 60 °C for 30 s). The sequences of primers were: NLRP3 *F*: 5′-TGA AGA GTG TGA TCT GCG GAA AC-3′; *R*: 5′-GAA AGT CAT GTG GCT GAA GCT GT-3′; NLRP1 *F*: 5′-GCC CTG GAG ACA AAG AAT CC-3′; *R*: 5′-AGT GGG CAT CGT CAT GTG T-3′; ASC *F*: 5′-AGT TGA TGG TTT GCT GGA TGC T-3′; *R*: 5′-GGT CTG TCA CCA AGT AGG GCT G-3′; Caspase-1 *F*: 5′-AAC CTT GGG CTT GTC TTT-3′; *R*: 5′-CAG GAG GGA ATA TGT GGG-3′; GAPDH *F*: 5′-AGA AGG CTG GGG CTC ATT TG-3′; *R*: 5′-AGG GGC CAT CCA CAG TCT TC-3′. The mRNA levels were calculated using the 2^−△△CT^ method. All experiments were performed in triplicate.

### Western blotting

After washed with ice-cold PBS, the cells were ultrasonically homogenized in a RIPA buffer and protease inhibitor cocktail, and then the homogenates were centrifuged at 12,000×*g* for 15 min at 4 °C. The protein concentration was quantified using a BCA protein assay kit (Pierce, Rockford, IL, USA) and the supernatants of homogenates were boiled at 100 °C in a laemmli sample buffer (Abcam, Cambridge, MA, UK) for 5 min. Samples were separated on a 10% SDS-PAGE and transferred to a polyvinylidene difluoride membrane (Millipore, MA, USA). Membranes were blocked with 5% (m/v) nonfat dry milk in 0.1% Tween 20 (TBS-T; 2 mmol/L Tris–HCl, 50 mmol/L NaCl, pH 7.4) for 2 h at room temperature and subsequently incubated overnight at 4 °C in the blocked buffer with NLRP3 antibody (Cat: 19771-1-AP; Proteintech, Chicago, USA), NLRP1 antibody (Cat: ab3683; Abcam, Cambridge, UK), ACS antibody (Cat: sc-514414; Santa Cruz, CA, USA), Caspase-1 p10 antibody (Cat: sc-514; Santa Cruz, CA, USA), and β-actin antibody (Cat: 8457S; Cell Signaling Technology, MA, USA), respectively. The membranes were washed with 0.1% Tween 20, and then treated with horseradish peroxidase-conjugated anti-mouse IgG (Abcam, Cambridge, UK) or goat anti-rabbit IgG H&L (HRP) (Abcam, Cambridge, UK) for 1 h at room temperature. After washing thrice with TBST, proteins were visualized with an electrochemiluminescence detection system and quantified by an image analysis system (Image J, MD, USA).

### Statistical analysis

All values are expressed as mean ± SEM. The statistical difference was analyzed using the SPSS (version 22.0). GO and pathway analysis were considered to be significantly enriched when hypergeometric *P*-value < 0.05. Quantitative analysis of our experiments was performed by Student's *t*-tests or one-way ANOVA. *P*-value < 0.05 was considered statistically significant.

## Results

### Sample preparation

**Fig. 1:**
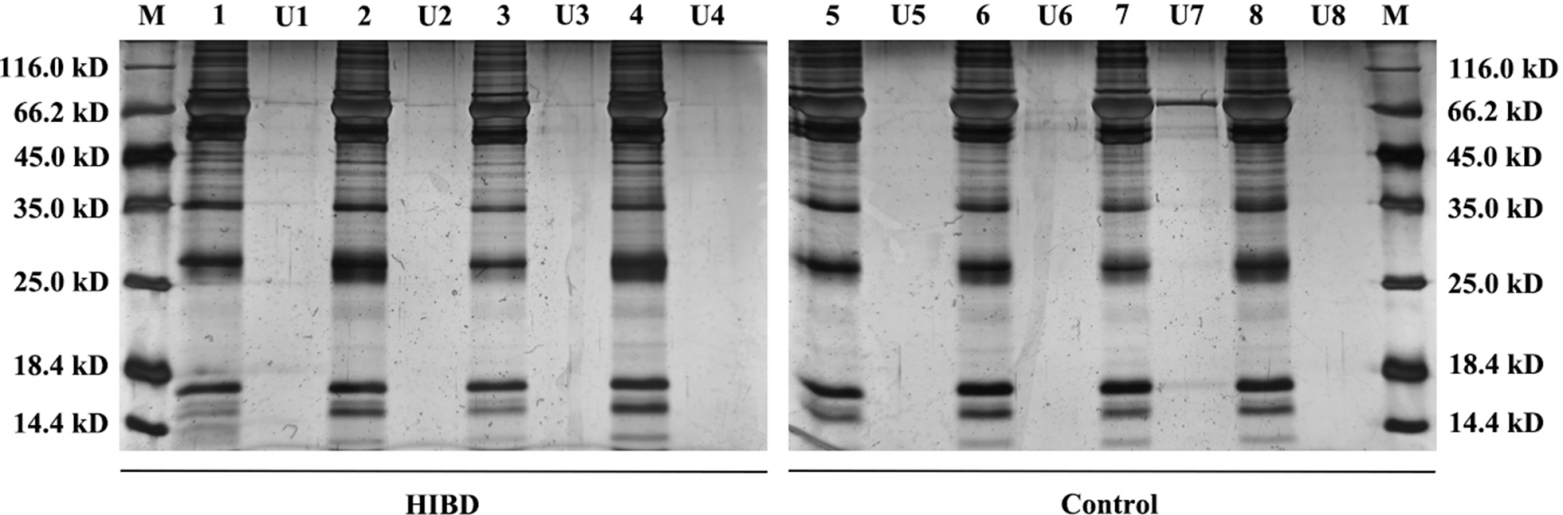
SDS-PAGE analysis of neonatal CSF samples before and after ultrafiltration treatment. 1–4: CSF samples of neonatal HIBD infants without any treatment; U1–4: CSF samples of neonatal HIBD infants after ultrafiltration; 5–8: CSF samples of controls without any treatment; U5–8: CSF samples of controls after ultrafiltration; M: protein marker

The peptidome constitutes only a minor part of the total protein contents of CSF. Because of the limited loading capacity of LC columns (< 1 μg), it is necessary to enrich the peptide fraction. Ultrafiltration using MWCO filters provides a simple means to remove high molecular weight proteins and retain a large part of peptides in the mass range of interest. The result of SDS-PAGE confirmed that most macromolecular proteins had been successfully removed (Fig. 1).

It was showed that lots of peptides of CSF bind to larger proteins and thus can be retained during the ultrafiltration step [18]. Therefore, urea, thiourea and DTT were used to pretreat CSF samples and dissociate peptides from carrier proteins prior to the ultrafiltration step. Salts had been removed by C18 solid phase extraction after ultrafiltration.

### Peptide identification

Peptides extracted from CSF of 4 newborns with HIBD and 4 normal controls were analyzed directly by LC–MS/MS. A total of 35 differentially expressed peptides (*P* < 0.05 and fold change > 1.5) from 25 precursor proteins were identified (Table 2). Compared with the control group, 1 peptide was up-regulated and 34 peptides were down-regulated in the HIBD group.

**Table 2:** Peptides that are differentially expressed in CSF between neonatal HIBD and normal control

Sequence	Mass	PI	Protein names	log2 (H/C)	*P*-value
Peptides up-regulated in HIBD (> 1.5 folds)					
GAGASSEP	978.6	3.85	Refilin-A (FAM101A)	2.7	1.59E−02
Peptides down-regulated in HIBD (> 1.5 folds)					
IAGYVTHLMK	1740.0	9.3	40S ribosomal protein S17 (RPS17)	− 2.4	4.38E−06
KIAFAITAIK	1987.3	10.81	40S ribosomal protein S18 (RPS18)	− 2.9	2.07E−06
YLYTLVITDKEK	2397.4	6.48	60S ribosomal protein L38 (RPL38)	− 13.8	3.17E−06
VAPEEHPVLLTEAPLNPK	2561.5	4.47	Actin, cytoplasmic 2 (ACTG1)	− 2.2	1.90E−04
TTGIVMDSGDGVTHTVPIYEGYALPHAILR	3791.0	5.36	Actin, cytoplasmic 2 (ACTG1)	− 3.8	9.57E−07
TTGIVLDSGDGVTHNVPIYEGYALPHAIMR	3804.0	5.36	Actin, gamma-enteric smooth muscle (ACTG2)	− 4.7	2.00E−06
VAPEEHPTLLTEAPLNPK	2867.7	4.47	Actin, gamma-enteric smooth muscle (ACTG2)	− 3.0	1.40E−04
FLSQPFQVAEVFTGHMGK	2646.4	7.55	ATP synthase subunit beta, mitochondrial (ATP5B)	− 8.0	2.18E−08
ISQMPVILTPLHFDRDPLQK	2955.7	7.55	GMP synthase [glutamine-hydrolyzing] (GMPS)	− 2.5	5.19E−06
SNYNFEKPFLWLAR	2392.3	9.3	GTP-binding nuclear protein Ran (RAN)	− 6.1	1.47E−06
IINEPTAAAIAYGLDKK	2699.6	6.49	Heat shock cognate 71 kDa protein (HSPA8)	− 5.4	2.52E−08
HSQFIGYPITLFVEK	2386.4	7.54	Heat shock protein HSP 90-alpha (HSP90AA1)	− 4.6	2.79E−08
HSQFIGYPITLFVEKER	2671.5	7.54	Heat shock protein HSP 90-alpha (HSP90AA1)	− 3.1	1.01E−05
NDEELNKLLGK	2183.3	4.43	Histone H2A.J (H2AFJ)	− 7.1	2.47E−04
VTIAQGGVLPNIQAVLLPK	2538.6	9.7	Histone H2A.J (H2AFJ)	− 2.8	4.00E−03
KTVTAMDVVYALKR	2506.5	10.24	Histone H4 (HIST1H4A)	− 3.0	8.15E−05
KTVTAMDVVYALK	2350.4	9.26	Histone H4 (HIST1H4A)	− 6.2	1.81E−05
VIQYLAVVASSHK	2022.2	9.3	Myosin-11 (MYH11)	− 5.4	3.90E−04
IVATKPLYVALAQR	2150.3	10.45	Polyadenylate-binding protein 3 (PABPC3)	− 11.8	1.66E−05
QILLYSATFPLSVQK	2315.4	9.3	Probable ATP-dependent RNA helicase DDX6 (DDX6)	− 3.9	1.27E−05
TLFVSGLPVDIKPR	2149.3	9.7	RNA-binding protein with multiple splicing 2 (RBPMS2)	− 6.2	3.34E−09
NILFVITKPDVYK	2461.5	9.26	skNAC (NACA)	− 9.4	6.45E−07
ILSGGLSYDTV	1166.6	3.75	Syntaxin-binding protein 5 (STXBP5)	− 2.5	2.30E−04
EMVELPLRHPALFK	2287.3	7.55	Transitional endoplasmic reticulum ATPase (VCP)	− 5.8	1.11E−05
LDHKFDLMYAK	2292.3	7.54	Tubulin alpha-1A chain (TUBA1A)	− 7.3	5.37E−06
MSATFIGNSTAIQELFKR	2620.5	9.7	Tubulin beta-2B chain (TUBB2B)	− 4.5	1.12E−07
YLTVAAIFR	1356.8	9.35	Tubulin beta-2B chain (TUBB2B)	− 4.3	1.67E−06
GPFGQIFRPDNFVFGQSGAGNNWAK	3391.7	9.7	Tubulin beta-2B chain (TUBB2B)	− 10.7	3.87E−08
KLAVNMVPFPR	1879.1	11.65	Tubulin beta-2B chain (TUBB2B)	− 7.7	1.86E−07
PFGQIFRPDNFVFGQSGAGNNWAK	3391.7	9.7	Tubulin beta-2B chain (TUBB2B)	− 7.5	1.41E−07
MAATFIGNSTAIQELFKR	2605.5	9.7	Tubulin beta-4A chain (TUBB4A)	− 4.4	4.18E−07
KEAESCDCLQGFQLTHSLGGGTGSGMGTLLISK	4351.3	5.54	Tubulin beta-4A chain (TUBB4A)	− 2.7	4.90E−04
LTDISVTDPEKYPHMLSVK	3083.7	5.52	U6 snRNA-associated Sm-like protein LSm2 (LSM2)	− 4.2	5.40E−03
GLLLLGHLTVDTY	1718.0	5.29	V-set and immunoglobulin domain-containing protein 4 (VSIG4)	− 2.9	2.10E−04

H: Peptide intensity (CSF of neonatal HIBD); C: Peptide intensity (CSF of normal controls)

### Characteristics of differentially expressed peptides

**Fig. 2:**
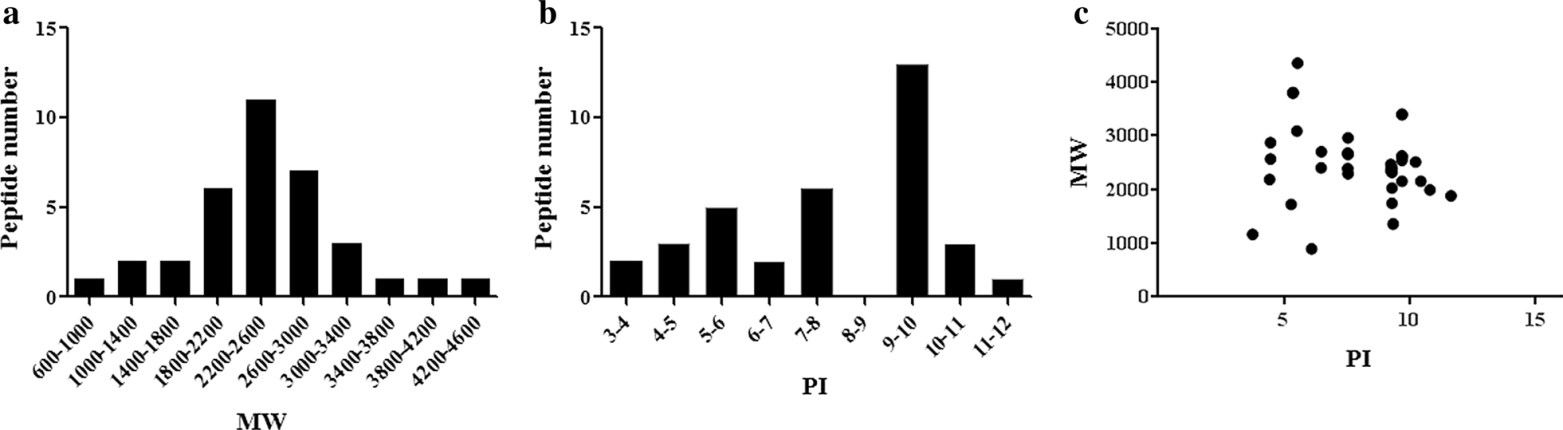
Characteristics of differentially expressed peptides. **a** The MW of most differentially expressed peptides ranges from 1800 to 3000 Da; **b** The PI of differentially expressed peptides most ranges from 9–10; **c** The distribution of MW versus PI of differentially expressed peptides. Points of these identified differentially expressed peptides are distributed around PI 10

We initially analyzed the molecular weight (MW) (Fig. 2a), isoelectric point (PI) (Fig. 2b) and the distribution of MW versus PI distribution (Fig. 2c) of differentially expressed peptides. The MW of most peptides ranges from 1800 to 3000 Da and the most widely distributed PI ranges from 9 to 10. Furthermore, the scatter plot of MW versus PI is shown in Fig. 2c. Points of these identified differentially expressed peptides are distributed around PI 10.

**Fig. 3:**
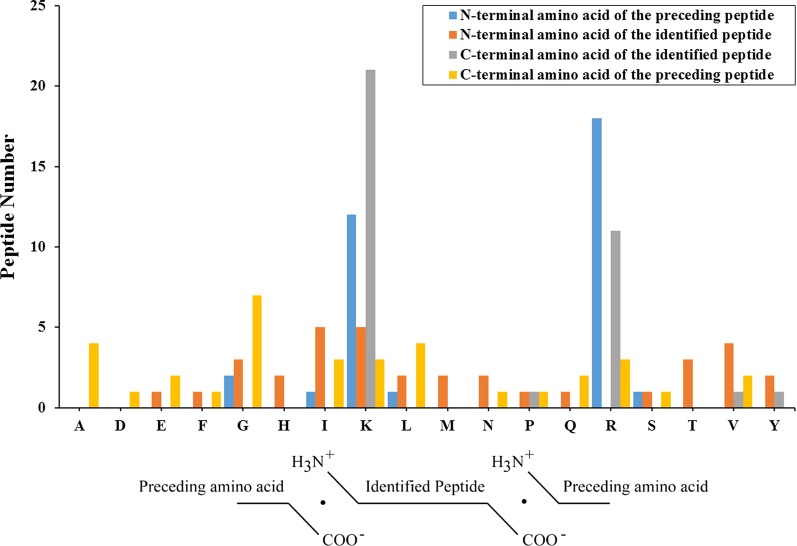
Cleavage sites in differentially expressed peptides. Arginine (R) and Lysine (K) were the most common cleavage sites of the N-terminal amino acid of the preceding peptide and C-terminal amino acid of the identified peptide. Glicine (G) was the most common cleavage site of C-terminal amino acid of the preceding peptide. Lysine (K) and isoleucinel (I) were the most common cleavage sites of N-terminal amino acid of the identified peptide

Next, we searched for cleavage sites in all differentially expressed peptides to study possible functional changes of CSF in HIBD. The cleavage sites of peptides are regular and we can study their rules to analysis the function of proteolytic enzymes (Fig. 3). Arginine (R) and Lysine (K) were the most common cleavage sites of the N-terminal amino acid of the preceding peptide and C-terminal amino acid of the identified peptide. Glicine (G) was the most common cleavage site of C-terminal amino acid of the preceding peptide. Lysine (K) and isoleucinel (I) were the most common cleavage sites of N-terminal amino acid of the identified peptide.

### Bioinformatics analysis

**Fig. 4:**
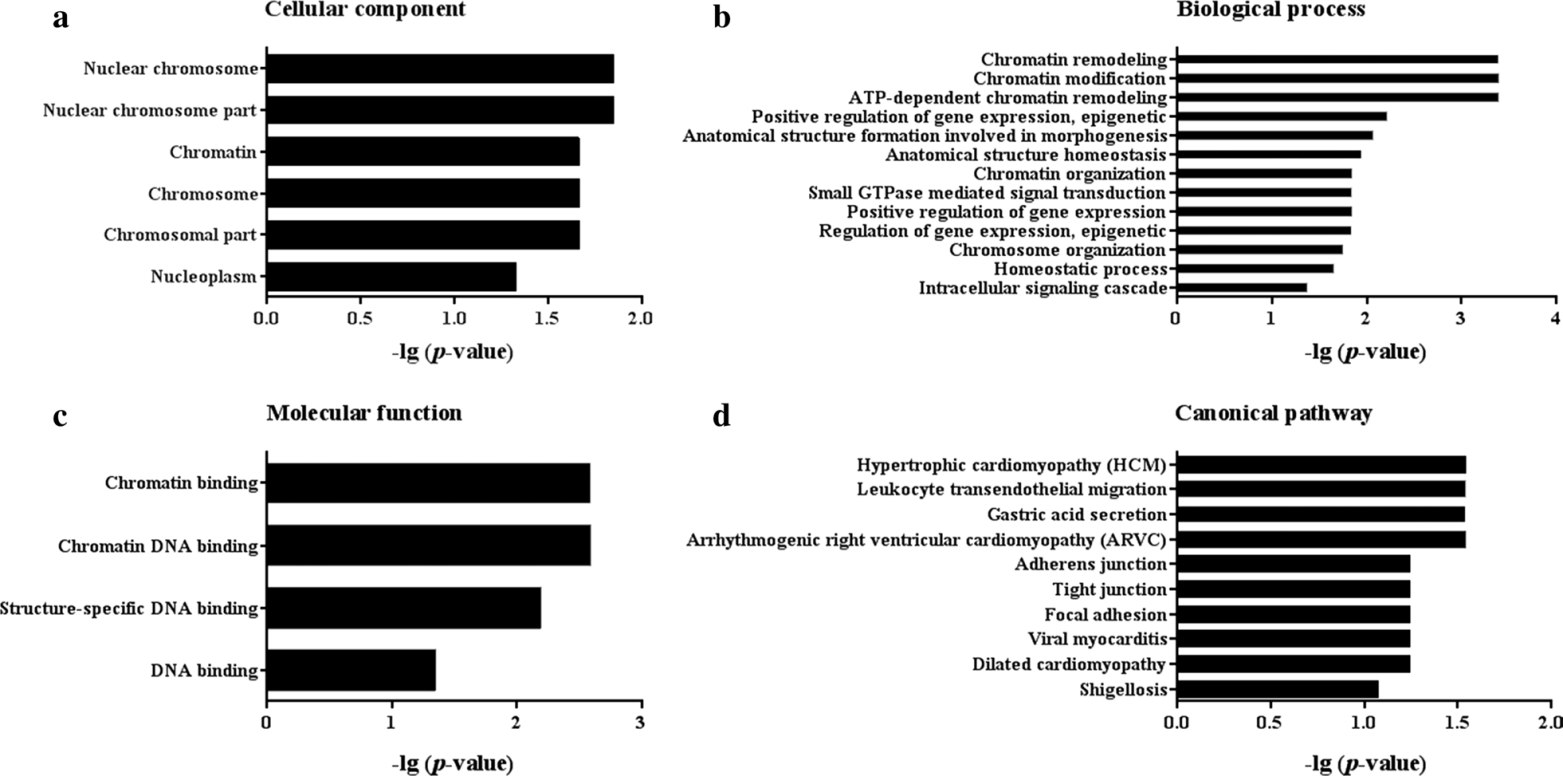
GO and KEGG analyses of peptide precursors. **a** The main cellular components of peptide precursors were nuclear chromosome, nuclear chromosome part, chromatin, chromosome, chromosomal part and nucleoplasm; **b** The most relevant biological processes were chromatin remodeling, chromatin modification, ATP-dependent chromatin remodeling and so on; **c** The molecular functions of peptide precursors mainly included chromatin binding, chromatin DNA binding, structure-specific DNA binding and DNA binding; **d** The most canonical pathways of peptide precursors were HCM, leukocyte transendothelial migration, gastric acid secretion, ARVC and so on

To preliminarily explore potential functions of these differentially expressed peptides in neonatal HIBD, GO and KEGG analyses were conducted based on their precursor proteins. The cellular components of peptide precursors were mainly as follows: nuclear chromosome, nuclear chromosome part, chromatin and so on (Fig. 4a). The most relevant biological processes were shown in Fig. 4b, including chromatin remodeling, chromatin modification, ATP-dependent chromatin remodeling and so on. The molecular functions of peptide precursors mainly included chromatin binding, chromatin DNA binding, structure-specific DNA binding and DNA binding (Fig. 4c). The pathway analysis showed that the most related pathways of these peptide precursors were hypertrophic cardiomyopathy (HCM), leukocyte transendothelial migration, gastric acid secretion, arrhythmogenic right ventricular cardiomyopathy (ARVC) and so on (Fig. 4d).

**Fig. 5:**
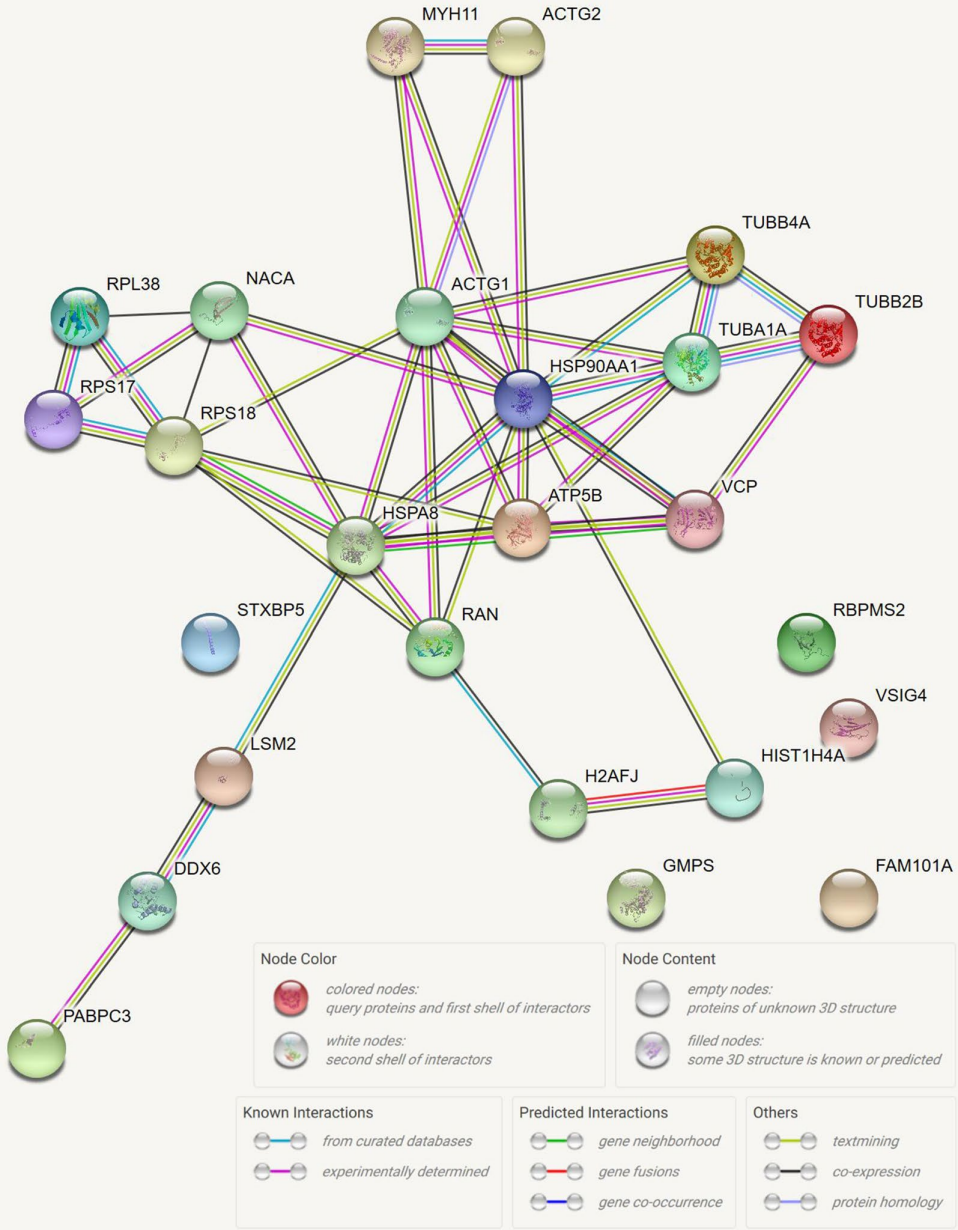
The protein–protein interaction network of peptide precursors. Each node represents all precursor proteins produced by a single, protein-coding gene locus. Edges represent protein–protein associations

In order to find important peptides that play key roles in HIBD, the functions of differentially expressed peptides and their precursor proteins were studied using STRING database and UniProt database. The protein–protein interaction (PPI) network of precursor proteins was shown in Fig. 5. We investigated the interaction of these precursor proteins and found that heat shock protein 90-alpha (HSP90α/HSP90AA1) was located in the network hub and exist in a complex relationship with other proteins.

### Effects of HIBDAP pretreatment on cell pyroptosis under OGD

**Fig. 6:**
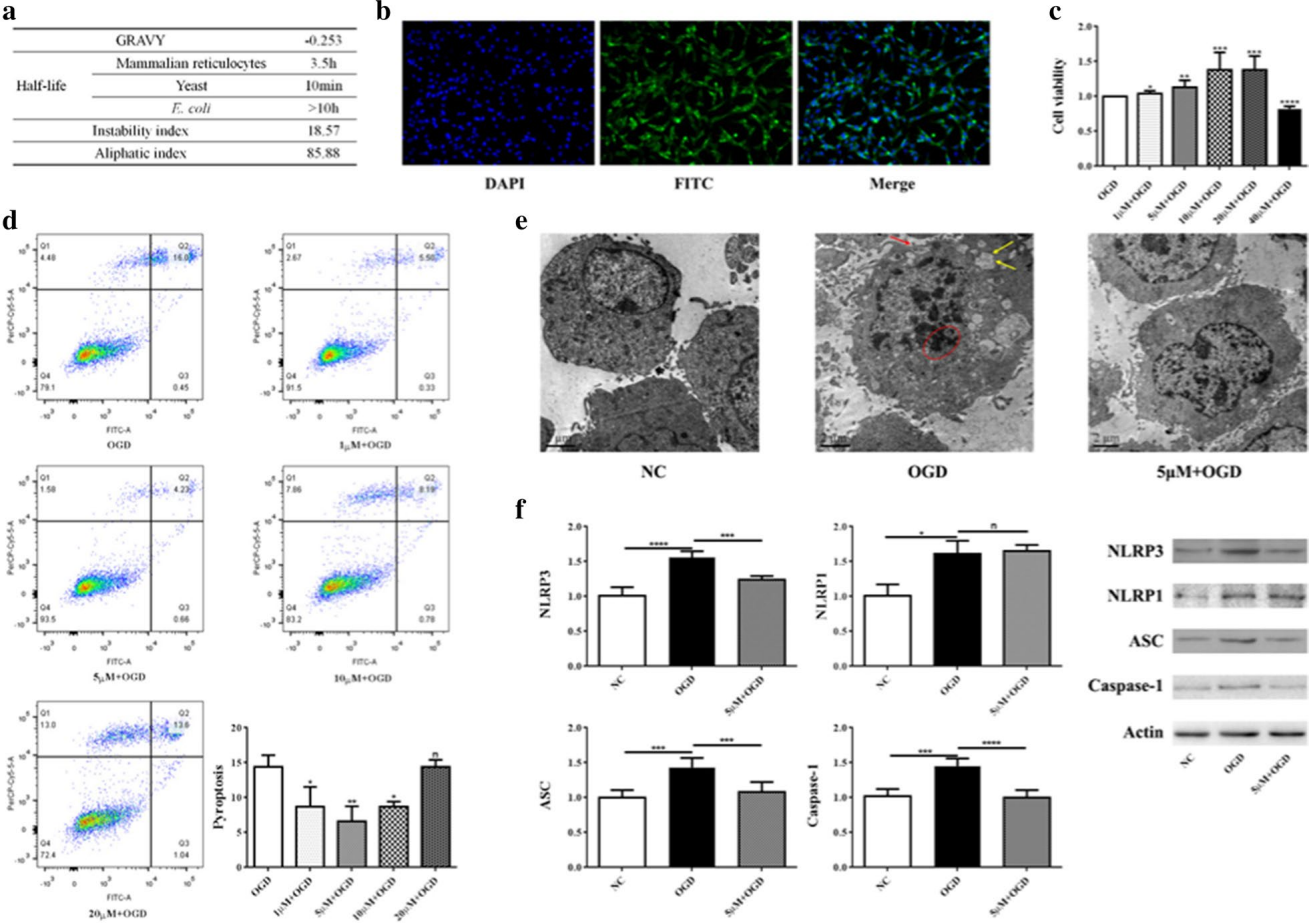
Effects of HIBDAP pretreatment on cell pyroptosis under OGD. **a** Bioinformatics analysis of HIBDAP; **b** FITC-labelled TAT-HIBDAP could successfully enter PC12 cells and further into the nucleus; **c** After HIBDAP pretreatment and 6 h of OGD treatment, the cell survival rate was significantly increased in PC12 cells pretreated with 1 μM, 5 μM, 10 μM and 20 μM HIBDAP except that of 40 μM group was significantly decreased; **d** The concentrations including 1 μM, 5 μM and 10 μM of HIBDAP significantly reduced pyroptosis of PC12 cells, except 20 μM had no effect; **e** Transmission electron microscopic examination showed that aggregated nuclear chromatin (the red circle), fuzzy cell membrane boundary (the red arrowhead), swollen endoplasmic reticulum (yellow arrowheads) in the OGD group (OGD), which could be alleviated by HIBDAP pretreatment (5 μM + OGD); **f** Compared with the control group (NC), the mRNA and protein expressions of NLRP3, NLRP1, ASC, and Caspase-1 were significantly increased in the OGD group (OGD) whereas HIBDAP pretreatment (5 μM + OGD) significantly suppressed their expressions except NLRP1 (^n^*P* > 0.05; *P < 0.05; **P < 0.01; ***P < 0.005; ****P < 0.001)

The 2671.5 Da peptide (HSQFIGYPITLFVEKER), one of the down-regulated peptides in neonatal HIBD, is a fragment of heat shock protein 90-alpha (HSP90α/HSP90AA1). We named it Hypoxic-Ischemic Brain Damage Associated Peptide (HIBDAP). As given in Fig. 6a, HIBDAP derives from the 210st to 226st amino acids of HSP90AA1 is a hydrophilic peptide with high stability and has a long half-life of 3.5 h in mammalian reticulocytes.

We coupled HIBDAP to the transduction sequence of the TAT protein, allowing for the delivery of peptide into cells. It was demonstrated that FITC-labelled TAT-HIBDAP could successfully enter PC12 cells and further into the nucleus (Fig. 6b). After HIBDAP pretreatment and 6 h of OGD treatment, we found that low concentrations of HIBDAP could increase the survival rate of cells revealed by results of CCK-8, except 40 μM had a toxic effect (Fig. 6c).

Pyroptosis is characterized by pore formation in the cell membrane resulting in cell swelling. Therefore, membrane-impermeant dyes, such as PI, stain pyroptotic cells but not apoptotic cells which cell membrane is intact. However, Annexin V could stain pyroptotic cells due to membrane pore formation and apoptotic cells due to exposed phosphatidylserine [19, 20]. Accordingly, pyroptotic cells were PI+/Annexin V+, while apoptotic cells were PI−/Annexin V+. As shown in Fig. 6d, safe concentrations of the peptide reduced pyroptosis of PC12 cells, except 20 μM had no effect.

Then, we evaluated morphological characteristics of pyroptotic cells which have unique morphological characteristics [21, 22] using transmission electron microscopy. PC12 cell under OGD was swollen and the boundary of cell membrane was blurred. Nuclear chromatin condensation and swollen endoplasmic reticulum were also observed in the OGD group. These morphological characteristics of the pyroptotic cell were alleviated by HIBDAP pretreatment (Fig. 6e).

Furthermore, we detected mRNA and protein expressions of the NOD-LIKE receptor (NLR) signaling pathway in PC12 cells pretreated with 5 μM HIBDAP under OGD. Compared with the control group, expressions of NLRP3, NLRP1, ASC, and Caspase-1 were significantly increased in the OGD group whereas HIBDAP pretreatment significantly suppressed their expressions except NLRP1 (Fig. 6f).

## Discussion

In this study, we identified differentially expressed peptides between the CSF of neonatal HIBD and controls using LC–MS/MS and found a total of 35 differentially expressed peptides, among which only 1 peptide was significantly up-regulated and 34 peptides were significantly down-regulated in the HIBD group (Table 2). The MW of most peptides was around 1800–3000 Da which confirmed that larger proteins were removed. The most widely distributed PI ranges from 9 to 10, which are in the alkaline group (Fig. 2).

Typical peptide precursors contain multiple sites for proteolytic processing that are usually pairs of basic amino acids, with Lys–Arg and Arg–Arg the most common. In our results, Lys–Arg was the most common cleavage site of N-terminal amino acid of the preceding peptide and C-terminal amino acid of the identified peptide (Fig. 3). The cleavage sites’ distribution of our peptidome is consistent with that of neuropeptides [23].

Most peptide precursors play roles on DNA binding in the nucleus and chromatin with the most related pathway including HCM, ARVC, dilated cardiomyopathy and so on (Fig. 4). Intriguingly, some significantly enriched pathways of precursor proteins were associated with cardiomyopathy. Studies have shown that molecular mechanisms of cardiomyopathy including dysregulated calcium flux, cardiomyocyte apoptosis and oxidative stress, which is also in the pathological process of neonatal HIBD [24].

Analysis of STRING database and UniProt database showed that precursor proteins of differentially expressed peptides were closely related to each other (Fig. 5). It was showed that the central protein is HSP90α/HSP90AA1, which means this precursor protein closely interrelated with other precursor proteins. A fragment of HSP90α/HSP90AA1 is the 2671.5 Da peptide (HSQFIGYPITLFVEKER), one of the down-regulated peptides in neonatal HIBD. Previous studies have shown that HSP90 is able to bind and stabilize the transcription factor hypoxia-inducible factor1-alpha (HIF-1α) [25–28], which acts as a cellular survival factor in response to tissue hypoxia [29–32]. Through the KEGG analysis, we found that HSP90α was involved in the NLR signaling pathway. NLRs are best known for their ability to form inflammasomes, which biochemical function is activating Caspase-1 to induce the pyroptosis by forming pyroptosome, a complex of oligomerized ASC molecules. Therefore, we speculated that this 2671.5 Da peptide, which we named it HIBDAP, may play roles in the mechanism of neonatal HIBD through pyroptosis, a novel mechanism of programmed cell death [33], by the NLR signaling pathway.

Results of bioinformatics analysis showed that HIBDAP with small molecular weight has high stability and long half-life, which revealed that it is suitable for the drug research. In accordance with other peptide drugs, the toxicity of HIBDAP increased with concentration. Our results showed that HIBDAP inhibited pyroptosis under OGD when its concentration below the harmful concentration (40 μM) except 20 μM. Beyond our expectations, groups of 10 μM and 20 μM had higher ratios of pyroptosis but also cell viability than the group of 5 μM. We speculated that the higher pyroptosis of 10 μM and 20 μM than that of 5 μM may due to its toxicity increasing. However, the number of living cells detected by CCK-8 can be influenced by various factors such as cell proliferation, pyroptosis, necrosis, apoptosis, autophagy and so on [34]. HSP90α, the precursor protein of HIBDAP, plays an important role in promoting cell proliferation [35, 36]. Therefore, groups of 10 μM and 20 μM had higher cell viability may because of the balance of pyroptosis and proliferation.

**Fig. 7:**
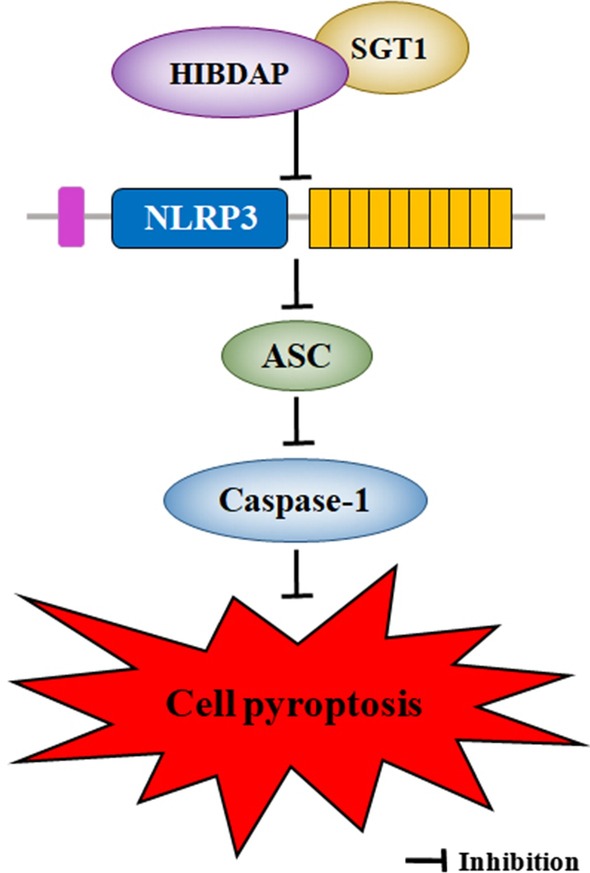
Diagrammatic drawing. HIBDAP inhibited pyroptosis under OGD by reducing expressions of NLRP3, ASC and Caspase-1 and may be a protective factor against neonatal HIBD through the cell pyroptosis mechanism

We further studied the mechanisms of effects of HIBDAP on cell pyroptosis using the concentration of 5 μM. It was revealed that HSP90 and SGT1 bind to NLRP3, one of NLR sensors, to inhibit its activation [37–39]. In addition to NLRP3, NLRP1 mediated inflammation is another classic pathway of pyroptosis [40]. Therefore, we detected expressions of NLRP3, NLRP1, ASC, and Caspase-1. The results of qRT-PCR and Western blotting showed that HIBDAP inhibited pyroptosis under OGD by reducing expressions of NLRP3, ASC and Caspase-1 except NLRP1. We presumed that, as same as the precursor protein Hsp90, HIBDAP interacts with NLRP3 instead of NLRP1 to suppress pyroptosis. We predicted HIBDAP may be a protective factor against neonatal HIBD through the NLRP3 inflammasome which is a required component in the mechanism underlying pyroptotic cell death (Fig. 7). The specific mechanism remains to be further studied.

The 2699.6 Da peptide (IINEPTAAAIAYGLDKK) originated from Heat shock cognate 71 kDa protein (HSPA8) was another down-regulated peptide in neonatal HIBD. HSPA8 is a major component of HSP70 family [41]. HSP70, another HSPs regulated by HIF-1α during hypoxia, appears to interrupt both cell death and immune responses which leads to improved neurological outcome [42]. Analysis of KEGG pathway revealed that HSPA8 is involved in the mitogen-activated protein kinase (MAPK) signaling pathway, which is involved in inflammatory responses [43] and neuronal death/apoptosis [44]. Therefore, we predict that this peptide may play roles in neonatal HIBD through the mechanism of cell death and inflammatory responses by the MAPK signaling pathway. However, it is unclear about detailed mechanisms and needs further studies.

Another interesting finding is that, of 35 differentially expressed peptides, 8 peptides come from the tubulin family, including Tubulin alpha-1A chain (TUBA1A), Tubulin beta-2B chain (TUBB2B) and Tubulin beta-4A chain (TUBB4A). The tubulin family is involved in the construction of cytoskeleton and is thought to be critical for the morphogenesis of axons and dendrites [45]. Recent studies revealed that tubulin isotypes caused a variety of neurological disorders [46] and tubulin-binding drugs can activate a component of the hypoxic adaptive response, specifically the stabilization of HIF-1α and its downstream targets [47]. Our results of the KEGG analysis found that Tubulin alpha and Tubulin beta were jointly involved in the regulation of gap junctions (GJs). In the central nervous system, GJs which were channels that connect the cytoplasm of cells form cellular syncytia and coordinate neural function. These channels have distinct functions within different cell types, and their expressions can change dramatically during neurodevelopment [48] and injury [49]. Therefore, these 8 peptides from the tubulin family are interesting targets for further study as potential pharmacotherapeutic methods.

In summary, we present the first data to demonstrate differentially expressed peptides in CSF of neonatal HIBD and controls. Several meaningful peptides such as HIBDAP may play significant roles in neonatal HIBD. The molecular mechanisms and biological functions of these peptides may provide new pathogenesis and therapeutic targets for neonatal HIBD.

## Data Availability

The datasets generated and analyzed during the current study are not publicly available due to privacy concerns but are available from the corresponding author upon reasonable request.

## Abbreviations

ACN: Acetonitrile
ARVC: Arrhythmogenic right ventricular cardiomyopathy
CARPs: Cationic arginine-rich peptides
CNS: Central nervous system
CSF: Cerebrospinal fluid
CT: Computed tomography
DTT: Dithiothreitol
EDTA: Ethylene diamine tetraacetic acid
FA: Formic acid
FITC: Fluorescein isothiocyanate
GO: Gene ontology
HCM: Hypertrophic cardiomyopathy
HIBD: Hypoxic-ischemic brain injury
HPLC: High-performance liquid chromatography
IDA: Information-dependent acquisition
KEGG: Kyoto encyclopedia of genes and genomes
LC–MS/MS: Liquid chromatography–tandem mass spectrometry
MAPK: Mitogen-activated protein kinase
MRI: Magnetic resonance imaging
MW: Molecular weight
MWCO: Molecular weight cut-off
NLR: NOD-LIKE receptor
OGD: Oxygen and glucose deprivation
PI: Isoelectric point
PMSF: Phenyl methyl sulfonyl fluoride
qRT-PCR: Quantitative real-time polymerase chain reaction
SDS-PAGE: Sodium dodecyl sulfate-polyacrylamide gel
SPE: Solid phase extraction
TAT: Transactivator of transcription
TEAB: Triethylammonium bicarbonate

## References

[1] Dixon BJ, Reis C, Ho WM, Tang J, Zhang JH (2015). Neuroprotective strategies after neonatal hypoxic, ischemic encephalopathy. Int J Mol Sci.

[2] Lawn JE, Cousens S, Zupan J (2005). 4 million neonatal deaths: when? where? why?. Lancet.

[3] Shetty J (2015). Neonatal seizures in hypoxic-ischaemic brain injury—risks and benefits of anticonvulsant therapy. Dev Med Child Neurol.

[4] Nemati H, Karimzadeh P, Fallahi M (2018). Causes and factors associated with neonatal seizure and its short-term outcome: a retrospective prognostic cohort study. Iran J Child Neurol.

[5] Vannucci SJ, Hagberg H (2004). Hypoxia-ischemia in the immature brain. J Exp Biol.

[6] Johnston MV, Fatemi A, Wilson MA, Northington F (2011). Treatment advances in neonatal neuroprotection and neurointensive care. Lancet Neurol.

[7] Natarajan G, Pappas A, Shankaran S (2016). Outcomes in childhood following therapeutic hypothermia for neonatal hypoxic-ischemic encephalopathy (HIE). Semin Perinatol.

[8] Higgins RD, Raju T, Edwards AD, Azzopardi DV, Bose CL, Clark RH (2011). Hypothermia and other treatment options for neonatal encephalopathy: an executive summary of the Eunice Kennedy Shriver NICHD workshop. J Pediatr.

[9] Lau JL, Dunn MK (2018). Therapeutic peptides: historical perspectives, current development trends, and future directions. Bioorg Med Chem.

[10] Soloviev M, Finch P (2006). Peptidomics: bridging the gap between proteome and metabolome. Proteomics.

[11] Fosgerau K, Hoffmann T (2015). Peptide therapeutics: current status and future directions. Drug Discov Today.

[12] Ji YB, Zhuang PP, Ji Z, Wu YM, Gu Y, Gao XY (2017). TFP5 peptide, derived from CDK5-activating cofactor p35, provides neuroprotection in early-stage of adult ischemic stroke. Sci Rep.

[13] Tu J, Zhang X, Zhu Y, Dai Y, Li N, Yang F (2015). Cell-permeable peptide targeting the Nrf2-Keap1 interaction: a potential novel therapy for global cerebral ischemia. J Neurosci.

[14] Edwards AB, Anderton RS, Knuckey NW, Meloni BP (2018). Perinatal hypoxic-ischemic encephalopathy and neuroprotective peptide therapies: a case for cationic arginine-rich peptides (CARPs). Brain Sci.

[15] Hölttä M, Zetterberg H, Mirgorodskaya E, Mattsson N, Blennow K, Gobom J (2012). Peptidome analysis of cerebrospinal fluid by LC-MALDI MS. PLoS ONE.

[16] Zougman A, Pilch B, Podtelejnikov A, Kiehntopf M, Schnabel C, Kumar C (2008). Integrated analysis of the cerebrospinal fluid peptidome and proteome. J Proteome Res.

[17] Knopman D (2001). Cerebrospinal fluid beta-amyloid and tau proteins for the diagnosis of Alzheimer disease. Arch Neurol.

[18] Shores KS, Knapp DR (2007). Assessment approach for evaluating high abundance protein depletion methods for cerebrospinal fluid (CSF) proteomic analysis. J Proteome Res.

[19] Edgeworth JD, Spencer J, Phalipon A, Griffin GE, Sansonetti PJ (2002). Cytotoxicity and interleukin-1beta processing following *Shigella flexneri* infection of human monocyte-derived dendritic cells. Eur J Immunol.

[20] Miao EA, Rajan JV, Aderem A (2011). Caspase-1-induced pyroptotic cell death. Immunol Rev.

[21] Vande Walle L, Lamkanfi M (2016). Pyroptosis. Curr Biol.

[22] Bian Y, Li X, Pang P, Hu XL, Yu ST, Liu YN (2020). Kanglexin, a novel anthraquinone compound, protects against myocardial ischemic injury in mice by suppressing NLRP3 and pyroptosis. Acta Pharmacol Sin.

[23] Zhang X, Che FY, Berezniuk I, Sonmez K, Toll L, Fricker LD (2008). Peptidomics of cpe(fat/fat) mouse brain regions: implications for neuropeptide processing. J Neurochem.

[24] Chen Z, Venkat P, Seyfried D, Chopp M, Yan T, Chen J (2017). Brain–heart interaction: cardiac complications after stroke. Circ Res.

[25] Liu YV, Baek JH, Zhang H, Diez R, Cole RN, Semenza GL (2007). RACK1 competes with HSP90 for binding to HIF-1α and is required for O2-independent and HSP90 inhibitor-induced degradation of HIF-1α. Mol Cell.

[26] Isaacs JS, Jung YJ, Mimnaugh EG, Martinez A, Cuttitta F, Neckers LM (2002). Hsp90 regulates a von Hippel Lindau-independent hypoxia-inducible factor-1α-degradative pathway. J Biol Chem.

[27] Katschinski DM, Le L, Schindler SG, Thomas T, Voss AK, Wenger RH (2004). Interaction of the PAS B domain with HSP90 accelerates hypoxia-inducible factor-1α stabilization. Cell Physiol Biochem.

[28] Baird NA, Turnbull DW, Johnson EA (2006). Induction of the heat shock pathway during hypoxia requires regulation of heat shock factor by hypoxia-inducible factor-1. J Biol Chem.

[29] Pichiule P, Agani F, Chavez JC, Xu K, LaManna JC (2003). HIF-1 alpha and VEGF expression after transient global cerebral ischemia. Adv Exp Med Biol.

[30] Chi NC, Karliner JS (2004). Molecular determinants of responses to myocardial ischemia/reperfusion injury: focus on hypoxia-inducible and heat shock factors. Cardiovasc Res.

[31] Lee JW, Bae SH, Jeong JW, Kim SH, Kim KW (2004). Hypoxia-inducible factor (HIF-1)alpha: its protein stability and biological functions. Exp Mol Med.

[32] Khan Z, Michalopoulos GK, Stolz DB (2006). Peroxisomal localization of hypoxia inducible factors and hypoxia-inducible factor regulatory hydroxylases in primary rat hepatocytes exposed to hypoxia-reoxygenation. Am J Pathol.

[33] Fernandes-Alnemri T, Wu J, Yu JW, Datta P, Miller B, Jankowski W (2007). The pyroptosome: a supramolecular assembly of ASC dimmers mediating inflammatory cell death via caspase-1 activation. Cell Death Differ.

[34] Galluzzi L, Vitale I, Aaronson SA, Abrams JM, Adam D, Agostinis P (2018). Molecular mechanisms of cell death: recommendations of the Nomenclature Committee on Cell Death 2018. Cell Death Differ.

[35] Ory B, Baud'Huin M, Verrecchia F, Royer BB, Quillard T, Amiaud J (2016). Blocking HSP90 addiction inhibits tumor cell proliferation, metastasis development, and synergistically acts with zoledronic acid to delay osteosarcoma progression. Clin Cancer Res.

[36] Zhou X, Wen Y, Tian Y, He M, Ke X, Huang Z (2019). Heat shock protein 90α-dependent B-cell-2-associated transcription factor 1 promotes hepatocellular carcinoma proliferation by regulating MYC Proto-Oncogene c-MYC mRNA stability. Hepatology.

[37] Gross O, Thomas CJ, Guarda G, Tschopp J (2011). The inflammasome: an integrated view. Immunol Rev.

[38] Mayor A, Martinon F, De Smedt T, Pétrilli V, Tschopp J (2007). A crucial function of SGT1 and HSP90 in inflammasome activity links mammalian and plant innate immune responses. Nat Immunol.

[39] Kadota Y, Shirasu K, Guerois R (2010). NLR sensors meet at the SGT1-HSP90 crossroad. Trends Biochem Sci.

[40] Chavarría-Smith J, Vance RE (2015). The NLRP1 inflammasomes. Immunol Rev.

[41] Kampinga HH, Hageman J, Vos MJ, Kubota H, Tanguay RM, Bruford EA (2009). Guidelines for the nomenclature of the human heat shock proteins. Cell Stress Chaperones.

[42] Kim JY, Yenari MA (2013). The immune modulating properties of the heat shock proteins after brain injury. Anat Cell Biol.

[43] Sulistyowati E, Lee MY, Wu LC, Hsu JH, Dai ZK, Wu BN (2018). Exogenous heat shock cognate protein 70 suppresses LPS-induced inflammation by down-regulating NF-κB through MAPK and MMP-2/-9 pathways in macrophages. Molecules.

[44] Kyriakis JM, Avruch J (2012). Mammalian MAPK signal transduction pathways activated by stress and inflammation: a 10-year update. Physiol Rev.

[45] Minoura I (2017). Towards an understanding of the isotype-specific functions of tubulin in neurons: technical advances in tubulin expression and purification. Neurosci Res.

[46] Magiera MM, Singh P, Gadadhar S, Janke C (2018). Tubulin posttranslational modifications and emerging links to human disease. Cell.

[47] Aleyasin H, Karuppagounder SS, Kumar A, Sleiman S, Basso M, Ma T (2015). Antihelminthic benzimidazoles are novel HIF activators that prevent oxidative neuronal death via binding to tubulin. Antioxid Redox Signal.

[48] Cina C, Bechberger JF, Ozog MA, Naus CC (2007). Expression of connexins in embryonic mouse neocortical development. J Comp Neurol.

[49] Freitas-Andrade M, Naus CC (2016). Astrocytes in neuroprotection and neurodegeneration: the role of connexin43 and pannexin1. Neuroscience.

